# Patient complaints on nurse job satisfaction in primary health care clinics, Ehlanzeni District, South Africa

**DOI:** 10.4102/phcfm.v17i1.5147

**Published:** 2025-12-09

**Authors:** Patrick N. Dlamini, Tebogo Tsele-Tebakang

**Affiliations:** 1 Department of Environmental Health, Faculty of Health Sciences, University of Johannesburg, Johannesburg, South Africa; 2 Department of Complimentary Medicine, Faculty of Health Sciences, University of Johannesburg, Johannesburg, South Africa

**Keywords:** job satisfaction, patient complaints, primary health care, primary health care nurses, nurses’ attitude, nurse burnout

## Abstract

**Background:**

South Africa has implemented several health improvements to strengthen primary health care (PHC). Despite that, there is an increasing number of patient complaints that may affect job satisfaction among healthcare providers, especially nurses.

**Aim:**

This study explored how patient complaints can impact job satisfaction of nurses in the PHC clinics, Ehlanzeni District, Mpumalanga, South Africa.

**Setting:**

Professional nurses from three PHC clinics in Ehlanzeni District, Mpumalanga, South Africa, were recruited.

**Methods:**

A qualitative, exploratory and descriptive design was utilised. Eleven professional nurses were recruited purposively at the three selected PHC clinics until saturation was reached. Semi-structured, individual interviews were conducted, transcribed verbatim and analysed thematically using Saldana’s coding methods.

**Results:**

Five major themes and sub-themes emerged: perceived staff attitudes and patient complaints, work environment stressors, emotional impact on nurses, communication challenges and recommended strategies for improvement. Primary health care nurses reported that patients’ complaints often stemmed from long waiting, insufficient staff and a lack of communication; however, these were reflected as staff failures, leading to reduced morale and confidence.

**Conclusion:**

Most reported patient complaints relate to negative staff attitudes, often compounded by systemic issues such as staff shortages, inadequate material resources and long waiting times. This study brings to the fore that patient complaints should be understood within the broader systemic context, as they can significantly influence nurses’ job satisfaction.

**Contribution:**

This study contributes empirical evidence to the under-researched area of the impact of patient complaints on nurses’ morale in the South African context.

## Introduction

Primary health care (PHC) serves as the first point of contact between patients and the healthcare system, focusing on basic treatment, preventive care, health promotion and the management of chronic diseases.^1^ In South Africa, PHC nurses play a significant role in providing frontline services, including patient assessment, treatment of ailments, management of chronic disease, health education and awareness.^2^ According to the General Household Survey (GH),^3^ seven out of ten households (approximately 73.1%) use PHC clinics in South Africa as their first contact for healthcare services. Additionally, the South African government has continued to implement programmes aimed at improving the quality of PHC clinics.^2^ Despite the positive initiatives, there is a mounting number of patient complaints, mainly because of undesirable staff attitudes and substandard nursing care, which have flagged concerns about competence and emotional impact on nurses.^4^

The South African National Patients’ Rights Charter was implemented in 2008 by the Department of Health (DoH) under section 27(1)(a) of the Constitution to guarantee patients’ rights to quality healthcare, including the right to complain about dissatisfaction with care provided.^5^ Patient complaints are an indication of interpersonal conduct of PHC nurses or failure within the healthcare system or a combination of the two.^6,7^ Patient complaints are a valued source to improve healthcare; however, they can weaken nurses’ morale and work commitment, leading to self-doubt, anxiety and burnout.^8,9^

These challenges are predominantly experienced in public PHC clinics, where nurses experience contextual difficulties such as insufficient staffing, high patient care demand and inadequate infrastructure and essential resources.^10,11^ Additionally, nurses must deal with the rising double burden of communicable and non-communicable diseases, which is a challenge to the country.^12^ These stressors contribute to moral distress, which impairs the well-being of nurses and causes job dissatisfaction.^8,13^

Long queues, system failures and weak referral systems further contribute to patient dissatisfaction, which frequently translates as complaints about negative staff attitudes.^14^ Despite working in a complex, stressful environment, nurses often lack organisational support platforms and opportunities for professional development. This could result in demoralisation and emotional withdrawal in high-demanding environments.^15,16^ South African studies have highlighted a correlation between burnout, work stress, absenteeism, shortage of staff and the job satisfaction of nurses.^17,18^ Job satisfaction represents the degree to which nurses appreciate their work, which is a significant aspect for both nurses and the institutions.

Considering South Africa’s ongoing efforts to implement National Health Insurance (NHI) and achieve Universal Health Coverage (UHC), it is critical to understand how patient complaints affect nurses’ well-being. While there are numerous studies on factors influencing patients’ complaints,^19,20,21^ comparatively minimal studies have explored how PHC nurses are affected by patient complaints.

The study is underpinned by the Job Demands-Resources (JD-R) model, which is a prominent occupational stress and well-being framework that distinguishes between job demands and job resources.^22^ In the context of this study, the conceptual framework draws upon the JD-R model to illustrate the dynamics influencing nurses’ satisfaction within PHC settings, where patient complaints are seen as job demands that elevate stress and diminish professionalism. Organisational support and work–life balance act as job resources that buffer these effects, ultimately influencing PHC nurse job satisfaction, a key outcome linked to well-being and healthcare service quality.

One of the key benefits of this framework is its ability to identify not only the direct relationship between the core constructs but also the underlying mechanisms (mediators) and influencing factors (moderators) that shape this relationship. By incorporating variables such as perceived stress and sense of professionalism, the framework captures the psychosocial processes through which complaints impact nurses’ mediating morale. Moreover, the inclusion of moderating variables like organisational support and work–life balance allows for a nuanced understanding of contextual and protective factors that may either buffer or intensify the effects of patient complaints.

## Research methods and design

### Research design and setting

This study used a qualitative, exploratory and descriptive design to understand how patient complaints affect nurse job satisfaction.

### Study setting

The study was conducted in three selected PHC clinics located within the City of Mbombela, a sub-district of the Ehlanzeni District in Mpumalanga province, South Africa. Ehlanzeni is the most populous district in the province with approximately 2.27 million residents (44% of the provincial population) and serves as the province’s economic hub. The district has 142 healthcare institutions, including 110 PHC clinics, 16 community health centres and 19 hospitals of various levels, with the City of Mbombela hosting the highest concentration of facilities (30 fixed facilities).^23,24^ The district is characterised by a high burden of disease, limited healthcare resources and significant workforce challenges. These clinics were purposively selected based on accessibility, patient volume and their representation of the PHC challenges common across the district.

### Research population

The target population (*n* = 65) for this study comprised professional nurses who were actively employed at PHC facilities in the Ehlanzeni District.

### Sample size and sampling

Purposive sampling was employed to select participants who met specific inclusion criteria, including being registered with the South African Nursing Council (SANC) as professional nurses or nurse practitioners, being permanently employed, having at least 5 years of experience working in a PHC setting within the district and having been directly exposed to patient complaints concerning staff attitudes. Data saturation was reached by the 10th interview, with the final interview confirming that no new information or insights were produced (*n* = 11). This level of saturation is consistent with empirical guidance, suggesting that homogenous study populations frequently reach saturation within 9–17 interviews.^25^ The rationale for ceasing data collection at this point aligns with rigorous qualitative methods and supports the credibility and trustworthiness of the findings.^26,27^

### Data collection

This qualitative study utilised a descriptive phenomenological approach to exploring the lived experiences of nurses. A semi-structured interview guide was specifically developed by the researcher to capture these experiences. The guide was informed by the study’s objectives, a thorough literature review and the conceptual framework. It was designed to be open-ended, allowing for in-depth exploration, and included probes focusing on key areas:

- Nurses’ perceptions of patient complaints regarding negative staff attitudes.
- The impact of these complaints on nurses’ job satisfaction and professional confidence.
- Coping mechanisms and support systems used by nurses.
- Recommendations for improving nurse well-being and patient–provider relationships.

The interviews started with an open question, ‘Please describe the procedure for patients to lodge complaints. To whom are complaints made directed?’ Followed by other questions:

- Can you describe an experience where you encountered a patient complaint regarding negative attitudes against professional nurses?
- How did this complaint affect you emotionally?
- Did this experience impact your professional confidence or sense of job satisfaction?
- How did you handle the situation emotionally?
- In your opinion, what factors in your work environment contribute to patient complaints about staff attitude?
- Can you elaborate on specific examples of how these factors might lead to such complaints?
- Can you suggest any interventions or changes that could improve the staff attitude of nurses and patients?
- What kind of support do you think nurses need to manage patient concerns about attitude effectively?
- Are there any specific communication strategies you think would help address these complaints?
- Is there anything else you would like to share about your experiences with patient complaints regarding staff attitude?

A pilot study was conducted with three nurses (who were not included in the final analysis) to test the interview guide. This pilot revealed the need to rephrase two questions for greater clarity and served to test the quality of the audio recording equipment and time management. The guide was refined based on these findings before the main study commenced.

Following institutional permission, professional nurses were purposively recruited from three PHC facilities in the Ehlanzeni district. Potential participants were approached in person and provided with a study information sheet. They were given at least 48 h to consider their participation. Written informed consent was obtained from all participants prior to their involvement in the study. All participants were informed of their right to withdraw at any time without penalty.

The data were collected from February 2025 to April 2025 through face-to-face interviews. All interviews were audio-recorded with a digital voice recorder after obtaining permission from the participants to ensure accurate data capture. Interviews were conducted in private consultation rooms within the premises of the clinics to ensure confidentiality and comfort. Interviews lasted for approximately 20 min – 40 min. Additionally, field notes were taken to document non-verbal cues and contextual details. Probing was used to elicit deeper insights.

To facilitate richer and more authentic responses, participants were given the option to be interviewed in their preferred language, including SiSwati, a language widely spoken by professional nurses in the district. All interviews conducted in SiSwati were later translated into English, with the accuracy of the translations verified. A total of 11 participants were interviewed. Data saturation was reached by the 10th interview, with the final interview confirming that no new information was emerging.

### Data analysis

Thematic analysis was employed using Saldaña’s coding techniques,^28^ which are well suited for exploring lived experiences. This involved first and second coding cycles of analysis: reading the transcripts to gain a holistic sense of the data, identifying meaning units, transforming the units into psychological language and synthesising the transformed units into themes. The data were managed and coded using Atlas.ti software (version 8.3), which facilitated systematic organisation and retrieval of codes and themes.

### Trustworthiness

The trustworthiness of the study was ensured through several strategies aligned with the criteria proposed by Lincoln and Guba.^29^ *Credibility* was established through member checking, where participants were invited to review and validate the transcriptions and emergent themes. *Dependability* was addressed by maintaining a clear audit trail of decisions made during the data collection and analysis phases. *Reflexivity* was upheld throughout the study, with the researcher continuously reflecting on personal biases and their potential influence on the research process.

### Ethical considerations

The ethical clearance was obtained from the University of Johannesburg’s Research Ethics Committee (REC) (REC-3157-2024). Permission was sought from the Mpumalanga Provincial Health Department REC and granted (MP_202410_011). Participation was voluntary, and nurses could withdraw at any stage without penalty. The study aimed to contribute positively to nursing well-being by giving participants a platform to share their experiences. Each participant was assigned a number for anonymity. Participants were monitored for distress during interviews, with referral pathways to counselling services available if needed. All eligible professional nurses meeting the inclusion criteria were invited fairly, ensuring equitable selection.

## Results

### Participant’s characteristics

Eleven individuals participated in this study. Females made up 9 (81.8%) of the participants, and males accounted for 2 (18.2%). Participants’ ages ranged from 29 years to 55 years, with a median age of 42. Most respondents, 7 (63.6%), spoke SiSwati, while 4 (36.4%) primarily spoke English during the interviews (Table 1). To ensure anonymity, all participant quotes are reported using a unique respondent number. All participants, 11 (100%), held a professional nurse qualification, and all 11 (100%) completed postgraduate diplomas in areas such as PHC, community nursing or midwifery.

**TABLE 1:** Demographic data of respondents.

Variables	Frequency	%
**Age (years)**		
20–30	1	9.1
31–40	3	27.3
41–50	5	45.5
51–60	2	18.2
**Gender**		
Male	2	18.2
Female	9	81.8
**Year of experience**		
5–15	6	54.5
16–25	5	45.5
**Language spoken**		
English	4	36.4
Siswati	7	63.6

Thematic analysis yielded five overarching themes and several sub-themes (Table 2). These themes encapsulate how professional nurses experience and interpret patient complaints related to negative staff attitudes and how these experiences affect their emotional well-being and job satisfaction.

**TABLE 2:** Themes and sub-themes.

Themes	Sub-themes
1. Patient complaints and perceptions of staff attitude	-
2. Workplace challenges and systemic barriers	2.1 High patient-to-nurse ratio 2.2 Long waiting times because of understaffing 2.3 Lack of basic resources 2.4 External interference
3. Emotional and professional impact on nurses	3.1 Feelings of frustration, helplessness and burnout 3.2 Loss of professional confidence 3.3 Emotional toll from systemic failures
4. Communication and nurse–patient relationships	4.1 Lack of explanations 4.2 De-escalation through empathy
5. Strategies for improvement and support interventions	5.1 Increasing staffing 5.2 Training on communication and conflict resolution 5.3 Management support and community engagement 5.4 Structured complaint resolution mechanisms

### Theme 1: Patient complaints and perceptions of staff attitude

This theme captured how nurses experienced patient complaints linked to their behaviour and communication. Patient complaints were frequently directed at perceived rudeness and dismissiveness, inadequate explanations and long waiting times, which nurses felt were often misinterpreted:

‘A patient accused a colleague of being dismissive … felt the nurse wasn’t taking their pain seriously, which led to a heated exchange.’ (Respondent 2, female, 38 years old, 12 years PHC experience)

‘Just last month, a patient complained because they had to wait for two hours to get a wound dressing.’ (Respondent 7, male, 44 years old, 14 years PHC experience)

‘Patients waiting for hours sometimes feel neglected, even though we’re doing our best.’ (Respondent 1, female, 45 years old, 18 years PHC experience)

### Theme 2: Workplace challenges and systemic barriers

Nurses attributed many complaints to systemic resource constraints rather than personal failure.

#### Sub-theme 2.1: High patient-to-nurse ratio

Nurses described how the high patient load prevented them from giving each patient sufficient attention, which patients sometimes misinterpreted as uncaring behaviour:

‘On a typical day, each nurse sees over 30 patients, which doesn’t allow us to spend enough time with each one.’ (Respondent 7, male, 44 years old, 14 years PHC experience)

#### Sub-theme 2.2: Long waiting times because of understaffing

Understaffing was highlighted as a major contributor to extended waiting times. Nurses reported that patients often grew frustrated during these delays, which escalated into complaints about staff attitude:

‘Waiting times at the clinic are three hours, and when patients don’t understand this, they get angry.’ (Respondent 10, female, 35 years old, 10 years PHC experience)

#### Sub-theme 2.3: Lack of basic resources

Resource shortages, such as a lack of medication, space or equipment, limited service delivery. Nurses expressed frustration that patients perceived them as negligent when systemic supply issues were beyond their control:

‘There are times when we run out of chronic medication, and patients think we are negligent when it’s a supply issue.’ (Respondent 3, male, 47 years old, 20 years PHC experience)

#### Sub-theme 2.4: External interference

Community leaders and management sometimes intervened in patient complaints in ways that undermined clinical authority, adding to stress and negatively affecting staff–patient relationships:

‘A local councillor and his members stormed the clinic, demanding explanations about patient complaints.’ (Respondent 9, female, 42 years old, 19 years PHC experience)

### Theme 3: Emotional and professional impact on nurses

Repeated complaints led to emotional exhaustion, self-doubt and reduced job satisfaction.

#### Sub-theme 3.1: Feelings of frustration, helplessness and burnout

Complaints, especially repeated ones, left nurses feeling unappreciated and emotionally drained. This fostered burnout and decreased morale across the team:

‘When a complaint arises, it feels like all the hard work you put in goes unnoticed.’ (Respondent 3, male, 47 years old, 20 years PHC experience)

‘I was frustrated because I knew I was doing my best under difficult circumstances.’ (Respondent 6, female, 36 years old, 11 years PHC experience)

#### Sub-theme 3.2: Loss of professional confidence

Nurses reported that frequent patient complaints led them to second-guess their clinical decisions, reducing confidence in their professional roles:

‘Such incidents make you second-guess your approach to patient care, even if you’re not directly involved.’ (Respondent 5, female, 41 years old, 13 years PHC experience)

#### Sub-theme 3.3: Emotional toll from systemic failures

Nurses often internalised complaints as personal criticism, even when systemic issues (such as shortages or waiting times) were the root cause. This emotional burden contributed to stress and reduced job satisfaction:

‘When patients complain, it feels like a personal attack, even when they’re just frustrated with the system.’ (Respondent 7, male, 44 years old, 14 years PHC experience)

### Theme 4: Communication and nurse–patient relationships

Poor communication was a key factor behind misunderstandings that led to complaints.

#### Sub-theme 4.1: Lack of explanations

Misunderstandings often occurred when patients were not adequately informed about systems such as booking or queue arrangements. These misunderstandings were frequently interpreted as unfair treatment:

‘If a patient does not understand the booking system, they feel discriminated against and assume we have favourites.’ (Respondent 8, female, 29 years old, 7 years PHC experience)

#### Sub-theme 4.2: De-escalation through empathy

Nurses noted that clear, empathetic communication and honesty about delays or challenges helped reduce complaints and rebuild patient trust:

‘Acknowledging a patient’s feelings upfront can de-escalate tension.’ (Respondent 6, female, 36 years old, 11 years PHC experience)

### Theme 5: Strategies for improvement and support interventions

Participants recommended key interventions to improve staff morale and patient satisfaction. These included increasing staff numbers, providing regular training in communication and stress management, enhancing management support with routine debriefing and community engagement and establishing clear complaint resolution mechanisms. Together, these strategies aim to address systemic challenges and foster a more supportive work environment.

#### Sub-theme 5.1: Increasing staffing

Nurses consistently emphasised the need for more staff to reduce waiting times and workload, thereby improving both patient satisfaction and job satisfaction:

‘We need more staff to reduce the workload and waiting times.’ (Respondent 2, female, 38 years old, 12 years PHC experience)

#### Sub-theme 5.2: Training on communication and conflict resolution

Ongoing professional development in communication, conflict resolution and stress management was viewed as essential for handling patient dissatisfaction more effectively:

‘Periodic workshops on communication skills and managing patient expectations would help.’ (Respondent 2, female, 38 years old, 12 years PHC experience)

#### Sub-theme 5.3: Management support and community engagement

Nurses recommended greater managerial involvement, regular debriefing sessions and community meetings to foster shared understanding of clinic challenges:

‘Community meetings should include nurses explaining clinic processes to reduce misunderstandings.’ (Respondent 11, female, 55 years old, 22 years PHC experience)

#### Sub-theme 5.4: Structured complaint resolution mechanisms

Nurses expressed the need for a formal, transparent complaint-handling system that would protect staff from unfair blame while ensuring patients’ voices were heard:

‘There should be an efficient complaints-handling system that ensures both staff and patients feel heard.’ (Respondent 6, female, 36 years old, 11 years PHC experience)

## Discussion of the findings

This study explored how patient complaints about negative staff attitudes impact the job satisfaction of professional nurses in PHC clinics in the Ehlanzeni District, Mpumalanga. The findings reveal a deeply intertwined relationship between patient dissatisfaction, systemic healthcare challenges and nurses’ emotional well-being. These findings align with global^30,31^ and South African studies,^32,33^ indicating that nurse job satisfaction is a multifaceted construct shaped not only by interpersonal dynamics but also by structural and resource-based conditions.

A central theme in this study is the misattribution of systemic healthcare challenges to individual nurses. While patients often perceive nurses to be rude or inattentive, nurses reported that these behaviours are often the result of overwhelming workloads, limited consultation time and high patient-to-nurse ratios. These findings are consistent with prior research by Van Der Heijden and colleagues^34^ and Havaei and MacPhee,^35^ which shows that nurses in understaffed environments are more likely to exhibit signs of stress and burnout, which can unintentionally affect patient interactions. This illustrates that patient dissatisfaction is symptomatic of broader systemic failings. Addressing staffing levels, enhancing communication protocols and managing patient expectations are critical to improving both patient experiences and nurse morale.

Respondents in this study did not dispute the occurrence of negative patient experiences but emphasised that their actions are frequently constrained by systemic barriers. This supports a study by Halcomb and colleagues,^36^ who argue that patient complaints in PHC are often symptomatic of deeper structural inefficiencies. Moreover, the JD-R model used in this study offers a useful lens, illustrating how excessive demands, without adequate organisational support, lead to emotional exhaustion and reduced professional engagement.

Nurses reported internalising patient complaints, often viewing them as a personal attack rather than systemic indicators. This emotional burden resulted in feelings of helplessness, demoralisation and a gradual decline in job satisfaction. Studies by Kwame^37^ and De Hert^38^ similarly highlight that emotional labour, when left unaddressed, contributes to psychological strain and staff attrition in healthcare settings.

The emotional toll is particularly acute in PHC contexts where nurses are often the sole health providers patients interact with. These findings resonate with the work of Gillespie and Reader,^4^ who describe how complaint systems, when not well managed, can damage staff morale and foster a culture of blame rather than learning. Awareness of these dynamics mandates that health leadership implement both demand-reduction strategies (e.g., adjusting workload) and resource-enhancement mechanisms (e.g., supportive supervision, training). Such actions can protect nurses from burnout and maintain job satisfaction.

Despite broader system challenges, respondents identified communication breakdowns as a common contributor to patient complaints. Misunderstandings were frequently rooted in a lack of clarity, rushed interactions or the inability to provide adequate patient education because of time constraints. This aligns with Karaferis and colleagues,^39^ who found that poor communication is a major driver of perceived staff negativity, even in technically competent care settings.

Importantly, this presents a modifiable area for intervention. Respondents suggested that regular training in conflict resolution, active listening and cultural sensitivity could mitigate patient dissatisfaction and improve interpersonal dynamics in PHC. The findings argue for investing in communication skills workshops and structured patient centredness. Enhancing communication can improve patient trust, reduce emotional strain on nurses and elevate the perceived quality of care.

The final theme captured practical interventions suggested by participants to address both systemic shortcomings and interpersonal tensions. Nurses overwhelmingly supported hiring additional staff to manage patient volumes and reduce burnout. This would allow for more patient-centred care and improved morale. Participants recommended structured training programmes to help manage patient expectations and de-escalate tense encounters. Suggestions included regular debriefing sessions, transparent leadership and public education campaigns to improve community understanding of clinic operations.

The findings underscore the importance of shifting from a blame-based complaint culture to one of system learning and psychological safety. Nurses advocated for structured complaint-handling systems that distinguish between legitimate misconduct and expressions of patient frustration stemming from systemic issues. These recommendations echo the conclusions of Novaes Neto and colleagues,^40^ who advocate for complaint systems that support healthcare workers rather than penalise them. The study also supports Kliestik and colleagues^41^ in suggesting that improving nurse morale is critical to achieving high-quality healthcare delivery and staff retention, especially in underserved areas like Ehlanzeni.

This study carries important implications for South Africa’s NHI ambitions. For the NHI to succeed, it must rest on the foundation of resilient, satisfied healthcare workers. Addressing nurse burnout, improving workplace conditions and reframing complaints as opportunities for systemic improvement are essential steps towards strengthening the PHC platform and achieving UHC.

### Job demands-resources model

The findings of this study demonstrate a strong conceptual alignment with the JD-R model. The model posits that high job demands, exemplified in this study by heavy workloads, repeated patient complaints and systemic shortages, can detrimentally affect employee well-being and job satisfaction, particularly when job resources are inadequate. Conversely, the model proposes that the provision of sufficient resources can mitigate the negative impact of such demands.

This study confirms the relevance of the JD-R model in the context of PHC settings in South Africa. Specifically, it highlights that patient complaints function as a significant job demand, which, when coupled with resource deficiencies and systemic inefficiencies, erodes nurse job satisfaction. The analysis further suggests that strengthening job resources, such as providing managerial and peer support, implementing targeted communication training and establishing structured complaints mechanisms, can act as a buffer against these demands. Consequently, the JD-R model offers a robust theoretical framework for the development of targeted interventions aimed at enhancing both nurse well-being and the quality of patient experiences within PHC facilities.

### Strengths and limitations

Conducted in the Ehlanzeni District, a region that typifies rural PHC challenges in South Africa, the study provides contextually relevant insights. Data saturation was reached by the 10th interview, with the final interview confirming that no new information or insights were produced, lending credibility to the thematic findings. Importantly, the study’s conclusions contribute to the broader discourse on health system strengthening under the *NHI Act*, which was signed into law in 2024.

The research was confined to three clinics within a single sub-district, which may limit the generalisability of the findings to other settings. The study also captured only the perspectives of nurses; incorporating views from patients and other healthcare workers might have yielded more comprehensive insights. As the data relied on self-reported experiences, there is potential for response bias. Additionally, the study reflects a cross-sectional snapshot in time and does not assess the long-term impact of patient complaints on job satisfaction.

### Recommendations

To improve nurse job satisfaction and patient care in PHC settings, several strategic interventions are recommended. Firstly, there is a critical need to invest in PHC nurse staffing to alleviate excessive patient loads and reduce burnout. Secondly, implementing regular training on communication and conflict resolution will equip nurses with the skills to manage difficult interactions, reduce incidences of perceived rudeness and foster a more empathetic approach to patient care. Such training prevents misunderstandings and enhances mutual respect, thereby improving patient satisfaction and strengthening nurse–patient relationships.^42,43^ Evidence further suggests that communication-focused interventions contribute to reduced patient complaints and improved job satisfaction among nurses, as they feel more confident in handling conflict situations.^42^ Establishing structured and transparent complaint-handling mechanisms can ensure that feedback is addressed constructively, protecting staff morale. Ultimately, it is crucial to promote collaboration among community leaders, patients and healthcare staff to align expectations and foster mutual trust within the healthcare system. Future research should expand to include patient perspectives alongside nurse experiences and explore longitudinal impacts of patient complaints on PHC staff retention and mental health.

## Conclusion

Most reported patient grievances emanate from nurses’ misconduct as compared to multiple systemic factors such as insufficient staffing, long waiting periods and inadequate essential resources. This study showed that nurses’ misconduct reflects systemic challenges. Moreover, nurses internalise the complaints, leading to self-doubt and reduced job satisfaction. Addressing these challenges is crucial in protecting the well-being of nurses and improving the quality of healthcare services.
